# Influence of the Plastic Deformation Process on the Residual Stresses and Hardness of an Al-5Mg Alloy

**DOI:** 10.3390/ma17143593

**Published:** 2024-07-21

**Authors:** Fayez Samara, Viorel Goanta, Bogdan Istrate, Layth Alkisswani, Corneliu Munteanu, Roxana Cosau

**Affiliations:** 1 Mechanical Engineering, Mechatronics and Robotics Department, Mechanical Engineering Faculty, “Gheorghe Asachi” Technical University of Iasi, 700050 Iasi, Romania; fayez.samara@student.tuiasi.ro (F.S.); layth.alkisswani@student.tuiasi.ro (L.A.); corneliu.munteanu@academic.tuiasi.ro (C.M.); elena-roxana.cosau@student.tuiasi.ro (R.C.); 2 Technical Sciences Academy of Romania, 26 Dacia Blvd., 030167 Bucharest, Romania

**Keywords:** residual stresses, hardness, plastic deformation, strain gauges, tensiometric rosette

## Abstract

The service behavior of ductile metallic materials, when they have previously undergone technological plastic deformation, depends on the deformation conditions. These are represented, among others, by the deformation rate, the process temperature, the applied pressures, and the introduced stresses, as well as other process variables. The investigation of the mechanical properties obtained after plastic deformation is an important means that contains two characteristics: on the one hand, to determine to what extent the parameters of the technological manufacturing process influence the main characteristics of the final component; and, on the other hand, on the basis of these characteristics, to analyze whether the component subjected to plastic deformation will be able to function reliably and safely. In the present work, an experimental study was made of the residual stresses developed and hardnesses obtained both in the immediate vicinity of a highly plastically deformed area and in an area previously obtained by rolling, without additional plastic deformation. For the determination of the residual stresses, the tensiometric rosette drilling method was used. By determining the same quantities in a non-plastically deformed area, significant changes in the values of the two quantities in the plastically deformed area were found. An increase in the maximum principal normal stresses by approx. 60 MPa and an increase in the Rockwel hardness by approx. 10 HRC was found. A sample was taken from the area under a plastic deformed circular shape, and was analyzed microscopically.

## 1. Introduction

Based on the plastic deformation mechanism, components were obtained whose subsequent properties may differ greatly from those of the original products. Significant variations in the mechanical, macroscopic, and microscopic characteristics of materials occur after the plastic deformation process. Of great importance is the prevention of defects arising from deformation, as well as the achievement of mechanical characteristics that lead to reliable and safe operation in service. Accumulation of damage during plastic deformation is the main cause of subsequent failures in operation, leading to reduced component life. Through plastic deformation of materials with predominantly ductile behavior, the dislocation movement is accentuated during the process, and is perpetuated and directed on slip planes depending on the direction of plastic deformation. Locking on internal obstacles or reaching the outer surface of dislocation trains leads to the nucleation of defects and their enlargement during the process of plastic deformation. Within this dynamic damage, the accumulation of residual stresses and strains occurs which, coupled with nucleated defects, will lead to the premature failure of the component [1]. In order to predict as accurately as possible, the behavior after plastic deformation, different models have been constructed based on macroscopic mechanical tests and microscopic structure tests, as follows: phenomenological constitutive, microscopic constitutive reflecting the microscopic deformation mechanism, and the artificial neural network constitutive model, respectively. Based on the existing research results, the advantages and disadvantages of the three constitutive models are compared and analyzed, respectively [2]. In establishing the constitutive model, in addition to considering the macroscopic deformation characteristics, the microscopic characteristics introduced to characterize the microscopic deformation of materials are also considered [3]. Plastic deformation results in a reduction in the grain size to the nanoscale, leading to an effective blocking of dislocation motion [4]. The reduction in grain size also results in a significant reduction in ductility due to the reduction in dislocation motion within small grain sizes. Unfortunately, there are not many studies that report on the monitoring of mechanical characteristics in the operation of components that have been obtained using plastic deformation. Moreover, simple characteristics obtained using static tensile testing of plastically deformed materials under different conditions are difficult to obtain due to the non-uniformity and asymmetry of the final components.

The ASTM E517-19 standard [5] is available, which provides the use of a special tensile test to measure the characteristic called Strain Ratio and graded with r for metal sheets that have been processed by severe plastic deformation. By obtaining and studying the values of this ratio, the aim is to highlight the anisotropy of the material from which the sheet is made. Through this report, it can also be highlighted if thinning and/or thickening of the sheets occurs as a result of the application of the technological process of plastic deformation. It is well known that this process introduces residual stresses, both at the surface and at depth, both uniform and non-uniform. The differences in the gradient of plastic deformation in different directions lead to the modification of the initial properties and the introduction of residual stresses [6,7,8,9,10,11,12,13]. A simple method by which the effect of plastic deformation on material characteristics is highlighted is represented by microhardness [14,15,16,17]. This must be carried out in the strongly plastically deformed area itself, in the area immediately adjacent to it, and in the area unaffected by the plastic deformation process. In one paper [14], the hardness distribution in different directions is presented in relation to the number of passes through severe lamination. When static tensile tests were performed on plastically deformed components in different directions, different characteristics were obtained. These characteristics, depending also on the type of plastic deformation, may not differ greatly, so that often the use of the term anisotropy may not be appropriate. In [18] a study made on the appropriate methods for the manufacture of nanostructured materials by severe plastic deformation, the results of the structural characterization of the materials obtained as a result of plastic deformation are presented; the study also investigated the less usual behavior during deformation, and obtained new properties. Considering the results obtained in this study on severe plastic deformation, a structural model was developed for the materials thus obtained. The effect on the structure and mechanical characteristics of samples made of an Al-Cu-Mg alloy with ultrafine grain was studied in [19]. It was possible to manufacture ultrafine-grained materials precisely by using severe plastic deformation techniques [20]. In this case, it was found, as above, that anisotropy is likely non-existent due to the plastic deformation adaptability of each grain. Also, for a high-pressure deformation process, a pure aluminum alloy (99.99%) was analyzed, consistent with the use of a two-step annealing process [21]. Samples were produced with large variations in structural parameters, including different dislocation density. If a low dislocation density is obtained, an additional hardening mechanism occurs, as a higher shear stress is required to activate the dislocation sources and direct them to suitable dislocation planes. In another work [22], thermomechanical processing of aluminum alloy AA2529 was performed, on the basis of which microstructures with different characteristics were obtained. Also in that paper, the emphasis was on the structural analysis of the material resulting from the technological process of plastic deformation, and was less on the resulting mechanical characteristics. For example, the influence of the aging process on Vickers hardness is not very clear. On the AZ31 and AZ61 alloys strongly plastically deformed by the ECAP (equal channel angular pressing) process, studies were carried out which, in addition to the obtained structure, some mechanical characteristics were compared in relation to the material before deformation. After applying the mentioned procedure, the following were obtained: a slight increase in microhardness, as well as for the yield limit and ultimate tensile strength. An interesting observation was that the yield limit decreased slightly after a higher number of cold presses. In any case, all of the mechanical characteristics were correlated with the deformed microstructure in the vicinity of the fracture surface.

In the framework of this paper, an experiment was designed and carried out by which the residual stresses were determined in an area strongly plastically deformed in an aluminum piece. The working method was by drilling the tensiometric rosette, established using the ASTM E837-20 standard [23]. The residual stresses were also determined in a non-plastically deformed zone, making comparisons with those determined in the plastically deformed zone. Also in the area in the immediate vicinity of the plastic deformation, the hardness variation along the length of the part was also determined, which was compared with the same variation obtained in the non-plastically deformed area. A microstructural analysis was made both in the deformed area in a circular shape and in the undeformed area. It can be noted that a series of cracks parallel to the width of the specimen appear, which normally lead to a decrease in its strength. Surprisingly, also here, Vickers indentation are performed, and their values increase in relation to the undeformed area.

## 2. Materials and Methods

For the following experiments and determinations, we had samples taken from an aluminum plate used in aviation. The aluminum plate was provided to us by a company that performs structural repairs in the aviation field. The data in Table 1 show the main component elements of the samples, and were obtained by means of a mass spectrometer.

**Figure 1:**
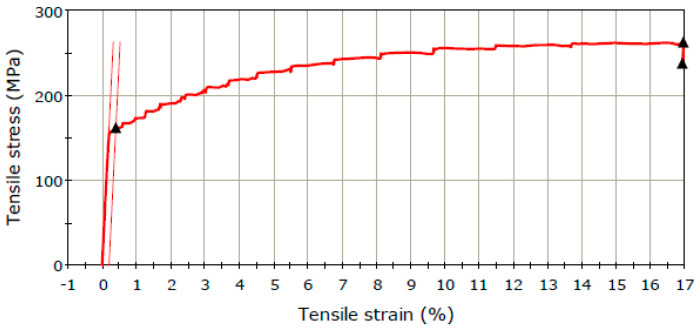
Characteristic stress−strain curve for the aluminum sample.

Within the present determinations, the specific deformations were measured and, on this basis, the residual stresses were determined on an aluminum plate, which was plastically deformed by pressing. The determinations of the residual stresses were carried out, for comparison, in a flat area located in the immediate vicinity of a severely plastically deformed area and in an area not plastically deformed. The method used to determine the residual stresses is by drilling the tensometric rosette. The characteristic stress–strain curve of the aluminum alloy, determined using the tensile test of a flat specimen, is shown in Figure 1.

The main mechanical characteristics emerging from the characteristic curve are as follows: Tensile strain at Yield, (Offset 0.2%): 162.3 MPa; Ultimate tensile strength: 262.7 MPa; Tensile strain at break: 0.17 mm/mm; and Energy at break: 49 J. For the subsequent determinations of the residual stresses from the specific strains provided by the strain gauges transducers, it is necessary to accurately determine the Poisson’s ratio and Young’s modulus.

**Figure 2:**
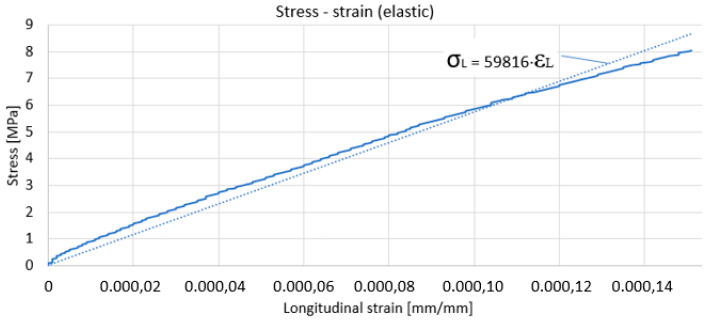
Longitudinal specific stress−strain variation for the reference sample.

**Figure 3:**
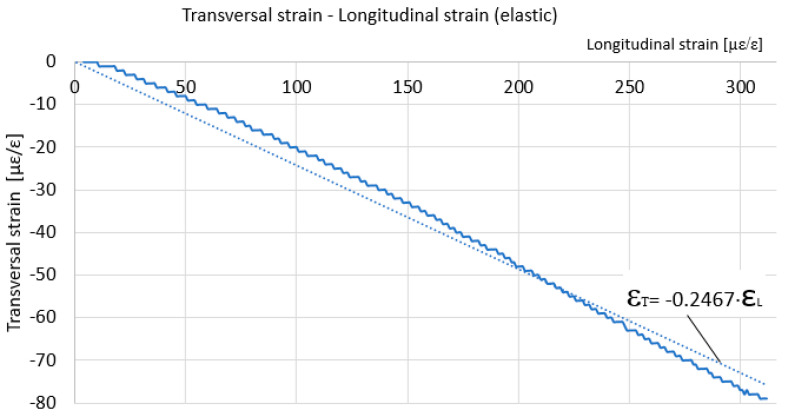
The variation of transverse strain versus longitudinal strain for the reference sample.

For these determinations, a tensometric transducer was mounted on a specimen made of the same material as that used to determine the residual stresses from which the specific deformations were taken when the specimen was subjected to tensile stress. The transducer used contains two strain gauges arranged in the form of †, with no electrical contact between the strain gauges. One of the strain gauge was arranged along the longitudinal direction—the stress direction—and the other strain gauge was aligned along the transverse direction of the specimen. The longitudinal specific strain was taken from the first strain gauge, and the transverse specific strain was taken from the second strain gauge. From the testing machine, the supplied data were taken, from which we selected the stress introduced into the sample in the calibrated section. The data acquisition rate was the same on the testing machine and the tensiometric bridge, one second, respectively. Thus, the data files from the testing machine and the strain gauge can overlap. The graph of variation of stress in relation to specific longitudinal strain, Figure 2, and the graph of variation of transverse specific strain in relation to specific longitudinal strain, Figure 3, resulted. It is mentioned that the loading was made only in the elastic domain. Under these conditions, the slope of the approximation line in Figure 2 represents Young’s modulus, and the slope of the approximation line in Figure 3 represents Poisson’s ratio.

**Figure 4:**
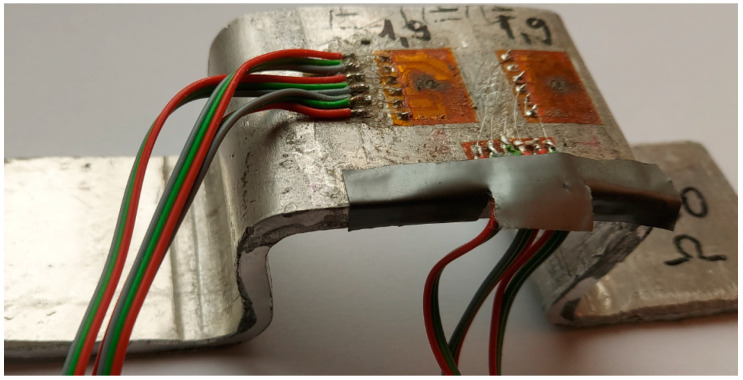
The sample used to determine the residual stresses.

As a result, the values of the two quantities that we need in the following for the calculation of the residual stresses are: Young’s modulus is 59,816 MPa (59.816 GPa), and Poisson’s ratio is 0.2467. Figure 4 shows the sample used to determine the residual stresses. It was severely deformed, the residual stresses being determined in the middle zone, undeformed, and in the lateral zone, subjected to significant plastic deformation. Type B strain gauge rosettes were glued near the plastically deformed zone, as in the middle of the specimen, where plastic deformation did not occur.

## 3. Procedure for Determining the Residual Stresses by the Drilling Method

### 3.1. Introduction within the Method Used

Residual stresses are present in almost all materials, often being higher immediately after the manufacturing process of the parts than after putting them into operation when it is possible, as a result of the working conditions to reduce them. Residual stresses can be introduced into the material of a part during manufacture or during its operation. If, from the design phase, they are not taken into account, the residual stresses can be an important factor that can lead to the failure or even to breaking of the component. This can occur especially in components that are subjected to alternating loads or work in a corrosive environment. There are also situations where residual stresses are beneficial, for example, when they are introduced into parts by the alice/sand blasting method. The method of determining the residual stresses used in this work is called the tensometric rosette drilling method. The tensometric rosette contains three resistive grids, with competing axes at one point, the method consisting in making a hole exactly at the point of concurrence of the grids’ axes. This method determines the variation with depth of the residual stresses near the surface of a material with isotropic and linear-elastic characteristics. This method is used, in particular, to determine residual stresses with depth where plane stress gradients are smaller. In experimental determinations, stresses may remain approximately constant with drilling depth (“uniform” stresses) or may vary significantly with depth (“non-uniform” stresses). The drilling stress measurement technique is a experimental method for determining residual stresses, which involves the removal of a small amount of material (by drilling a countersunk hole with dimensions D_0_ = 1.5 ÷ 3 mm and z ≈ 1.2·D_0_), which usually do not affect the operation of the part. The hole method can identify residual stresses in the plane near the surface of the material of the part [24]. The method provides localized measurements that indicate residual stresses within the boundaries of the drilled hole. This method of determination is applied in cases where the behavior of the material is linear-elastic. In certain situations, it can be said that the drilling method is semi-destructive because the damage is localized, the resulting hole being small in size, not affecting the continued operation of the respective part. As a result, this method should be used in cases where either the workpiece will no longer be used or when the small hole drilled will not significantly affect the operation of the part. There are other methods that cause substantial damage of the part on which the residual stresses are determined [25].

### 3.2. Working and Calculation Methods for Determination Residual Stresses

**Figure 5:**
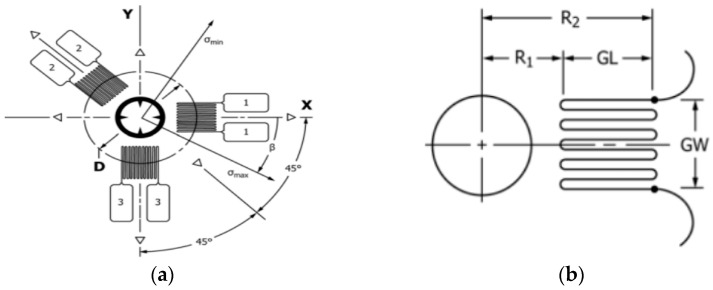
Schematic geometry of a typical system with three counter-clockwise (CCW) strain gauges. (**a**) Rosette layout. (**b**) Detail of a specific strain gauge [23].

As a test location within the workpiece, two flat and uniform surfaces were chosen: one in the immediate vicinity of the plastically deformed area, and the other in a non-plastically deformed area, which do not contain irregularities. Two tensiometer rosettes, each with three resistive grids, as shown in Figure 5, were glued onto the plastically deformed sample in two measuring zones. The hole is drilled at the point of intersection of the grid axes, the residual stresses around the hole being partially released as the hole advances. The drilling is performed in steps, with the specific deformations measured using a strain gauge.

Residual stresses existing before drilling into the material are determined based on the stresses released by drilling leading to strains. Attenuated strains depend on the residual stresses that existed in the material inside the hole. Residual stresses are determined using mathematical relationships from linear elastic theory. In the case of the uniform stress state, the deformation at the surface, measured after drilling, is given by the following relation:

(1) $$\epsilon =\frac{1+v}{E}\bar{a}\frac{\sigma_{x}+\sigma_{y}}{2}+\frac{1}{E}\bar{b}\frac{\sigma_{x}-\sigma_{y}}{2}\cos2\theta +\frac{1}{E}\bar{b}\tau_{xy}\sin2\theta$$

where:

- *ν* is Poisson’s ratio;
- *σ_x_, σ_y_*, and *τ_xy_* are the plane stresses corresponding to the x and y directions in Figure 5a;
- *E* is the longitudinal modulus of elasticity (Young);
- *θ* is the angle between the direction of the desired deformation and the X axis;
- $\bar{a}$ and $\bar{b}$ are calibration constants that are dimensionless and almost independent of the material.

**Figure 6:**
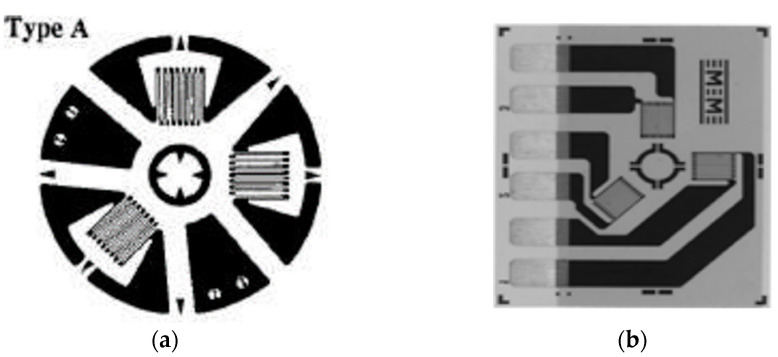
Tensiometer rosette used for drilling method: (**a**) general scheme of a rosette type A; (**b**) Vishay micro-measurements rosette type CEA-XX-062UL-120.

The determination of the specific strains released during each drilling step provides sufficient information to calculate the stresses *σ_x_, σ_y_*, and *τ_xy_* within each drilling step. Based on these stresses, the normal principal stresses *σ_max_* and *σ_min_* can be obtained as well as the orientation angle of the normal maximum principal stress in relation to the x axis, Figure 5a. The relaxed strains are mainly influenced by the residual stresses near the surface. Interior stresses are influenced by certain factors that lead to their decrease in depth from the surface. Thus, borehole measurements can only evaluate stresses near the surface. For a “thick” part, if a borehole depth smaller than the part thickness is to be used, the part thickness must be at least 1.2D for a type A rosette, Figure 6a. Vishay micro-measurements rosette type CEA-XX-062UL-120 was used for the determinations, Figure 6b. The thickness of the part was 6 mm, the drilling depth was 2 mm, and D is 1.92 mm.

### 3.3. Method of Strain Determination and Instrumentation Used

A rosette comprising three simple strain gauges is used, Figure 6b. In relation to the point of intersection of the axes of the resistive grids, they are arranged circularly. Two of the grids are arranged at 90 degrees to each other, the third grid being placed on the bisector of the mentioned 90-degree angle, but outside it. The three directions are noted as follows: (1)—the reference direction, which usually lies on the longitudinal direction of the piece; (2)—the direction at 135 degrees in relation to the direction (1); and (3)—perpendicular to the reference direction, Figure 6b. Direction (1) is identified with the measurement direction x, with direction (2) being located 90 degrees from it, counterclockwise.

#### 3.3.1. Drilling Equipment

**Figure 7:**
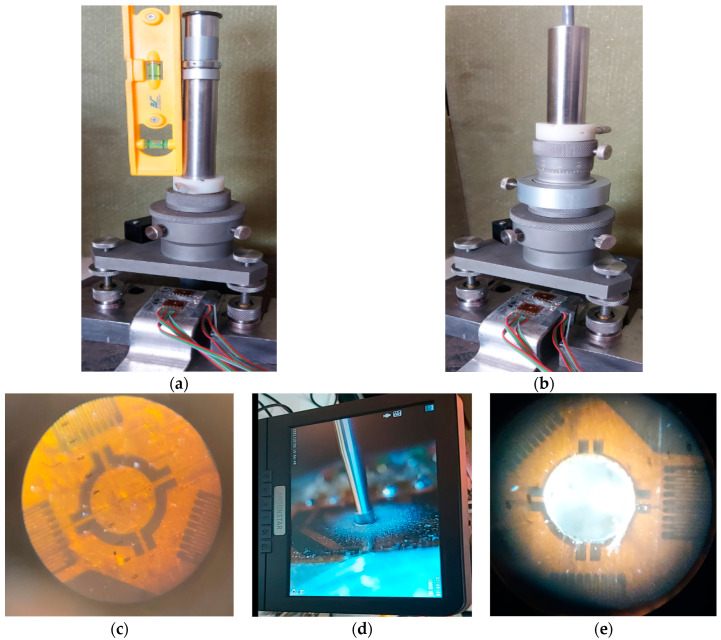
Device used and working steps: (**a**) clamping and centering the magnifying device on the workpiece; (**b**) turbine and feed devices; (**c**) visualizing the centering with the optical device; (**d**) drilling in the center of the transducer; (**e**) hole resulting after drilling.

A special device was used which is equipped with a centering magnifier and a high-speed air turbine and with special cutters to execute a hole in the workpiece in a controlled manner, Figure 7a,b. The device is capable of drilling a hole concentrically aligned with the strain gauge circle, Figure 7c, which is provided with alignment devices for this purpose. The device is capable of controlling the depth of the hole to ±0.004 D. Figure 7d illustrates the drilling mode in the center of the transducer at the intersection of the three strain gauge axes. The hole in the specimen, Figure 7e, was made with a high hardness milling cutter (made of metal carbides) with a diameter of 1.6 mm driven by a compressed air turbine at speeds up to 40,000 rpm. The flat-bottomed hole is drilled in the center of the rosette to a depth of Z = 1.2∙D_0_ = (1.2∙1.6) = 1.92 mm ≈ 2 mm. An inverted conical milling cutter was used for easy removal of the chipping debris. Inverted taper cutters have a maximum diameter at the end face and taper slightly towards the shank. When drilling takes place, the front of the cutter chips the material and releases it into the reverse taper area, with clearance between the tool and the hole wall. This minimizes contact between the cutter and the side surface of the hole, and minimizes the possibility of introducing additional residual stresses.

To begin with, the supporting material of the tensometric rosette will be removed, also with the help of the milling cutter, thus removing any sign of intersection of the axes or the existence of the centering circle. As a result, until this moment, the turbine with the cutter should already be very well centered and fixed. The drilling depth here will not be considered, and the point where the cutter has reached will be considered as the “zero” point. After the milling cutter touches the base material, the values indicated by the tensometric bridge will be balanced to zero on each of the three channels. The turbine starts and advances the drill by 0.2 mm at 0.2 mm intervals, stopping the drill feed to read the specific strains from the strain gauge.

#### 3.3.2. Calculation Method of Residual Stresses

The relations for the calculation of residual stresses as described below. Using the strains taken from the strain gauges, *ϵ*_1_, *ϵ*_2_, *ϵ*_3_, the different combinations of strains will be calculated based on the following relationships [23]:

(2) $$p=\left(\epsilon_{3}+\epsilon_{1}\right)/2$$

(3) $$q=\left(\epsilon_{3}-\epsilon_{1}\right)/2$$

(4) $$t=\left(\epsilon_{3}+\epsilon_{1}-2\epsilon_{3}\right)/2$$

Based on these three combinations of strains p, q, and t, the values for P, Q, and T, which represent combinations of the stresses in the plane state, are calculated as follows:

(5) $$P=\frac{\sigma_{y}+\sigma_{x}}{2}=-\frac{Ep}{\bar{a}\left(1+v\right)}$$

(6) $$Q=\frac{\sigma_{y}-\sigma_{x}}{2}=-\frac{Eq}{\bar{b}}$$

(7) $$T=\tau_{xy}=-\frac{Et}{\bar{b}}$$

where P = isotropic (equi-biaxial) stress, Q = shear stress at 45°, and T = shear stress.

In relations (5), (6), and (7) appear the quantities $\bar{a}$ and $\bar{b}$, which are the calibration constants. Their numerical values are calculated using the data in Table 1 according to the procedure described below.

Stresses σ_x_, σ_y_, and τ_xy_, in the Cartesian plane, are calculate using the following relations:

(8) $$\sigma_{x}=P-Q$$

(9) $$\sigma_{y}=P+Q$$

(10) $$\tau_{xy}=T$$

The principal (main) stresses, *σ_max_* and *σ_min_*, are calculated using the following relation:

(11) $$\sigma_{max},\sigma_{min}=P\pm \sqrt{Q^{2}+T^{2}}$$

The angle *β* between the direction of the maximum principal normal stress and the direction of the x axis (see Figure 5a) is calculated with the following relation:

(12) $$\beta =\frac{1}{2}\tan^{-1}\frac{-T}{-Q}$$

By calculating the angle *β* using the arctan function with a single argument, it can provide a deviation of ±90 degrees. In the present determinations, the value of the angle *β* was adjusted by adding or subtracting the value of 90 degrees, as the case may be, in order to place b in the appropriate range. 

#### 3.3.3. The Procedure for Determining the Calibration Constants $\bar{a}$ and $\bar{b}$ in Relation to the Data Taken from the Experiment

To determine the values of the quantities P, Q, and T with the help of relations (5), (6), and (7), it is necessary to know the constants $\bar{a}$ and $\bar{b}$. From standard E837, the values for the constants $\bar{a}$ and $\bar{b}$ at different ratios between the hole depth and the mean diameter of the tensiometer transducer grids are taken, which, for transducers of type CEA-06-062UL-120, used in this experiment, was 5.13 mm. The diameter hole measured after drilling is D_0_ = 1.9 mm. Thus, the ratio D_0_/D = 0.37 was obtained. From the mentioned standard, the closest ratio was chosen, namely 0.35, for the determination of the constants $\bar{a}$ and $\bar{b}$, see Table 2. In our experiment, we do not have exactly the same h/D ratios as provided by the standard; therefore, it was necessary to interpolate the results provided by the standard to obtain the constants $\bar{a}$ and $\bar{b}$ for the existing ratios in our experiment.

**Figure 8:**
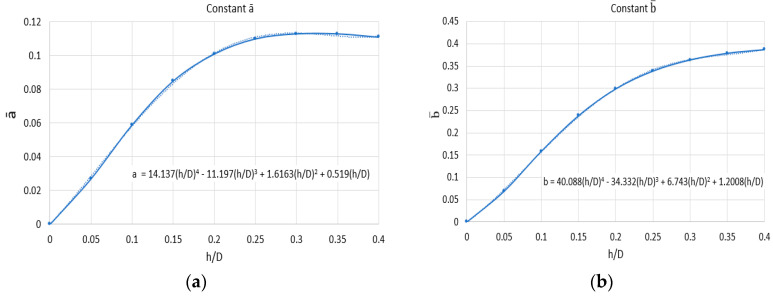
Variation of constants $\bar{a}$ and $\bar{b}$ as a function of the ratio h/D: (**a**) constant $\bar{a}$; (**b**) constant $\bar{b}$.

**Figure 9:**
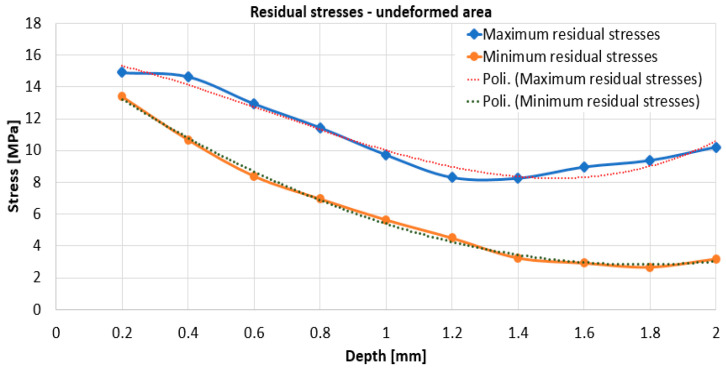
Variation of maximum and minimum residual stresses for the undeformed area.

Using the data from Table 1, the graphs in Figure 8 and Figure 9 were drawn. 

The expressions determined by approximating the points obtained with polynomials of order 4 are used to calculate the constants $\bar{a}$ and $\bar{b}$ for the *h/D* ratios used in our experiment, Table 3.

## 4. Results Obtained for the Residual Stresses on the Depth of the Plastically Deformed and Undeformed Zones

**Figure 10:**
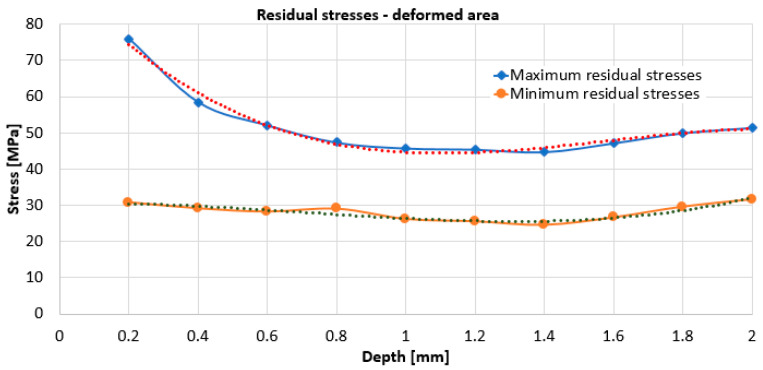
Variation of residual stresses, maximum and minimum, for the deformed area.

Using relations (2 ÷ 7), the values for the specified sizes were determined. With their help and using relation (11), the maximum and minimum residual stresses were calculated. For both working zones, i.e., for the plastically deformed marginal zone and for the central non-plastically deformed area, it can be seen, from Figure 9 and Figure 10, that the highest residual stresses, both maximum and minimum, are obtained for the 0.2 mm depth. 

With increasing working depth, the residual stresses decrease. For the undeformed area, Figure 9, the variation of the maximum stresses is with initial decrease in the 0.2–1.2 mm zone followed by a slight increase up to 2 mm depth. For the undeformed area, the variation of minimum stresses is initially decreased in the area 0.2–1.8 mm. For the undeformed zone, the maximum residual stress, at 0.2 mm depth, is relatively small, respectively, 15 MPa. For the deformed zone, Figure 10, the variation of the maximum stresses decreases in the zone depth of 0.2–1.4 mm, followed by a slight increase.

**Figure 11:**
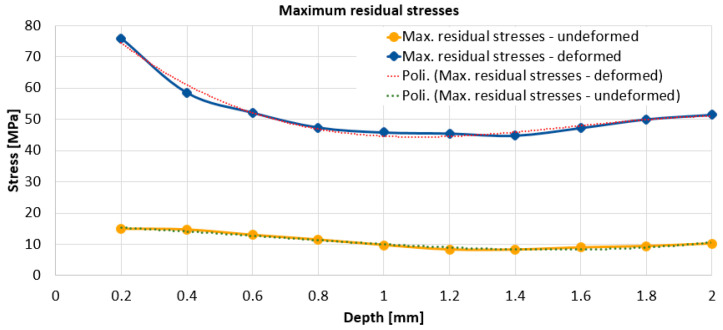
Variation of maximum stresses for the two zones, deformed and undeformed.

From Figure 9 and Figure 10, it can be seen that the highest value of the maximum residual stresses in the undeformed zone is approx. 15 MPa, while the value of the maximum residual stresses in the deformed zone is approx. 76 MPa. From Figure 11, it can be seen that the maximum values of the residual stresses, for each depth, are higher for the plastic deformed zone than for undeformed zone of the specimen. 

In any case, the relaxation of residual stresses occurs when, for certain working depths, their values stabilize on a range of variation. As a result, for the deformed zone, it can be estimated that the maximum residual stress value of approx. 45 MPa is representative, while for the non-deformed zone, we can consider the maximum residual stress value to be approx. 10 MPa.

**Figure 12:**
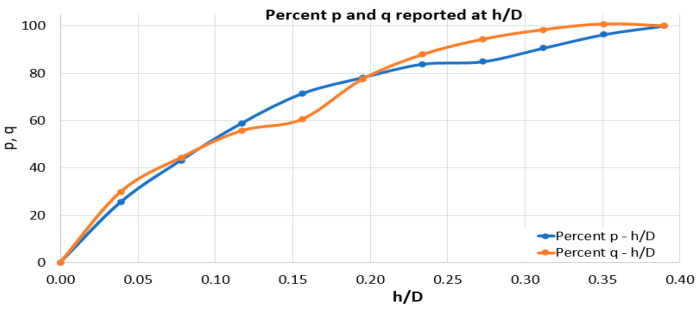
Variation of percentage *p*, *q*, with respect to *h*/*D*.

To validate the values obtained for the residual stresses, the variation graphs for the quantities p and q in relation to *h*/*D* are drawn, Figure 12. 

It can be seen that the points obtained, on the basis of which these graphs were plotted, lie on curves similar to those in the standard. There are no large deviations (greater than 63%), and, as a result, the data obtained can be validated by indicating a relative uniformity of stresses across the thickness of the material.

## 5. Microstructural Analysis of Plastically Undeformed/Deformed Area 

**Figure 13:**
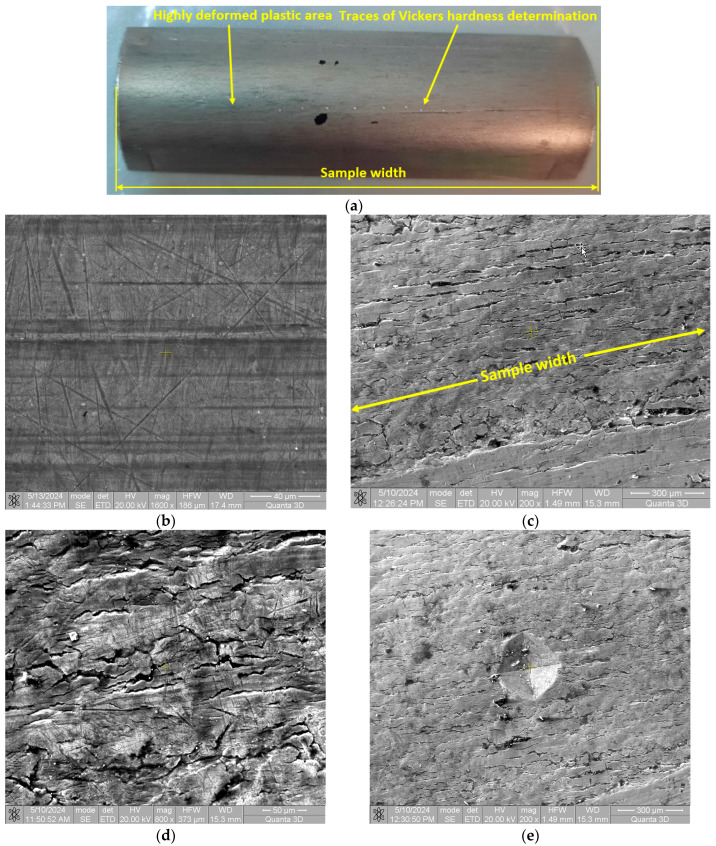
Surface appearance: (**a**) sample used for microscopic views; (**b**) in the undeformed area; (**c**) in the deformed area—200×; (**d**) in the deformed area—16,000×; and (**e**) in the Vickers indentation area.

From the immediate vicinity of the plastically deformed area where macroscopic residual stresses were determined, a sample was taken and microstructurally analyzed, Figure 13a. The images in Figure 13c–e are taken from the upper, strongly plastically deformed circular zone. Figure 13b shows the appearance of the surface from an undeformed area where only traces of scratches or marks from the rolling process can be seen. Figure 13c shows the image of the strongly plastically deformed area (upper circular area) at 200× magnification. Damage to the material can be seen, mainly in the form of longitudinal cracks running parallel to the width of the sample, as shown in Figure 13a.

Figure 13d shows an image of the heavily deformed area at 800× magnification. Here, it can be seen that in addition to longitudinal cracks (parallel to the specimen width), there are also small cracks at different angles to the specimen width. In Figure 13e, an image at 220× power is observable with the Vickers indentation trace in the plastically deformed area; see Figure 13a. The Vickers indentation, due to the ductility of the material, does not introduce additional cracks in the diagonal extension of the indentation trace. As a result, the results obtained and provided below are worthy of consideration for characterizing the degree of plastic deformation of the material. 

## 6. Hardness Variation in Deformed and Non-Deformed Plastic Zones

**Figure 14:**
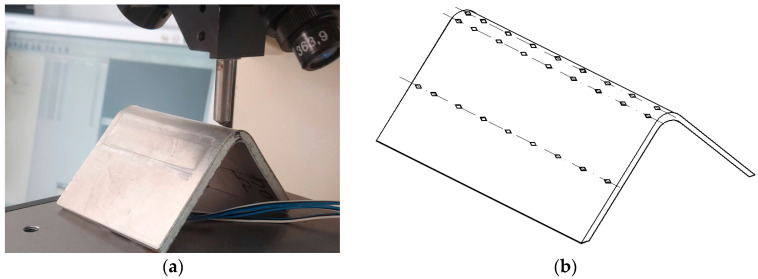
Vickers indentation in the curved deformed zone. (**a**) Image of the indent mode. (**b**) Configuration of the three ident areas.

Hardness is defined as the property of a material to resist the penetration of a harder body into its surface layers. Hardness is a measure of a material’s resistance to plastic deformation, which can also be related to yield strength. The mechanical properties of a material, usually derived from the stress–strain characteristic curve obtained using uniaxial tension, can be evaluated using indentation [26,27]. As found, a connection can be made between the plastic deformation of a material and its hardness determined after that plastic deformation [26]. For example, if a sample of a metallic material is stressed in tension to the area where plastic flow occurs in the material, the hardness does not remain the same as in a material undergoing only elastic deformation. The tests were carried out using a Vickers microhardness tester capable of acquiring and processing digital images. In order to see whether hardness varies with the degree of plastic deformation, three rows of indentations were made for the sample in Figure 14. 

**Figure 15:**
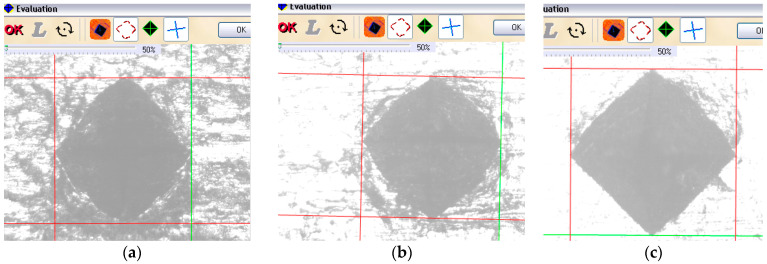
Vickers indentation traces in the three zones: (**a**) in the curved plastically deformed zone; (**b**) in the zone in the immediate vicinity of the crack; and (**c**) in the non-deformed zone.

The first row of indentations was made exactly in the plastically deformed area in the middle of the exterior arc of the circle resulting from plastic deformation, Figure 14a. The second row of indentations was made in the immediate vicinity of the plastically deformed area, but on the flat surface, Figure 14b. The third set of indentations was made on a flat surface away from the plastically deformed area. Figure 15a–c show the appearance of the traces resulting from the indentations in the three areas. In the highly plastically deformed area, Figure 15a, the surface is found to contain cracks and traces of excessive plastic deformation, i.e., shaded areas of the surface. In the near planar area, Figure 15b, such traces are also found, but are much less. In the non-deformed area, Figure 15c, the surface remains white, apart from the area in the vicinity of the indentations which also undergoes plastic deformation. 

**Figure 16:**
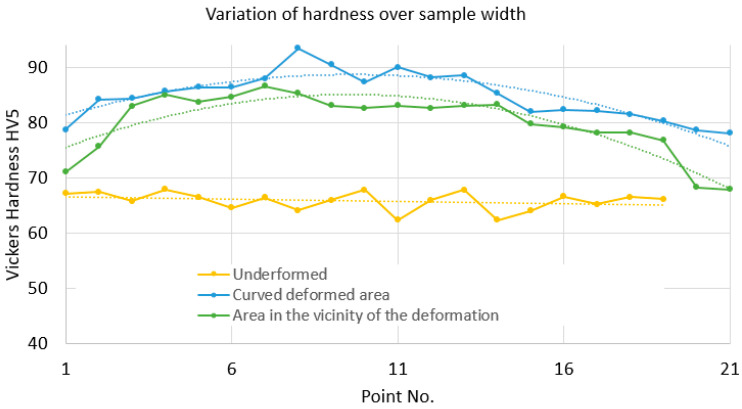
Hardness variation graph for three zones: deformation in the circular/curved zone, the zone in the vicinity of the deformation, and the undeformed zone.

Based on the data obtained with the Vickers HV5 indentation, the graphs in Figure 16 showing the microhardness values in the three areas specified above were plotted. It can be seen that the highest values of the hardnesses are obtained in the arc area, the average value being 84.9 HV5. Somewhat unexpectedly, in the immediate vicinity of the plastic deformation, on the first plane zone existing after the curvature, close values were obtained, the average hardness value being 80.08 HV5. For both zones, lower hardness values are obtained towards the ends of the sample, which implies that here the plastic deformation was not as high as in the center. Obviously, in the undeformed area, the microhardness values were lower, the average value being 65.88 HV5. Also in this area, there was a certain constancy in the values obtained for microhardnesses. 

As a result, it can be concluded that the hardness value can be a measure of the degree of plastic deformation [26]. 

## 7. Conclusions 

In this paper, two experiments were designed, implemented, and carried out to determine the residual stresses and hardnesses in a sample subjected to severe plastic deformation. It was expected that where we have severe plastic deformations, producing large distortions of the crystal lattice, we would also have high residual stresses. In the experiment, we used the tensiometric rosette drilling method to determine the residual stresses. Two tensor rosettes of type A were glued to the test sample; see Figure 6. All the steps specified by the standard [23] for such a determination were carried out, with the specification that some adaptations regarding the determination of the $\bar{a}$ and $\bar{b}$ constants were necessary. The results obtained lead to the conclusions described below.

For both working zones, i.e., for the plastically deformed marginal zone and for the central non-plastically deformed zone, from Figure 8 and Figure 9 it can be seen that the highest residual stresses, both maximum and minimum, are obtained for the 0.2 mm depth. With increasing working depth, the residual stresses decrease. For the undeformed zone, the variation of the stresses, maximum and minimum, is with an initial decrease in the range of 0.2–1.2 mm followed by an area with constant or slightly increasing stresses. For the deformed zone, the variation of the maximum stresses initially decreased in the 0.2–1.4 mm zone followed by a slight increase, while the minimum stresses, after the 0.2 mm depth, remain approximately constant, however, with a slight increase towards the end, as do the maximum stresses. 

The highest value of minimum residual stresses in the undeformed zone is approx. 13 MPa, and the highest value of minimum residual stresses in the deformed zone is approx. 32 MPa. The highest value of maximum residual stresses in the undeformed zone is approx. 15 MPa, and the highest value of maximum residual stresses in the deformed zone is approx. 76 MPa. Although the residual stresses with one of the tensometric rosettes were determined in the flat area in the immediate vicinity of the curved area and were strongly plastically deformed, from the hardness determination, if we consider the hardness value as a measure of the degree of plastic deformation, we find that even here the plastic deformation was significant. Obviously, it is possible that in the curved area, the residual stresses are somewhat higher, and by equivalence with the hardness values, they could reach approx. 83.75 MPa. It can be concluded that the minimum and maximum values of the residual stresses for each depth are higher for the plastic deformed zone than for the central undeformed zone of the plate. Residual stresses in the undeformed zone of 16 MPa are usual for the technological rolling process to which the material has been subjected, while the residual tensile stresses of 76 MPa (or 80.57 MPa) for the deformed zone are quite high, if one takes into account a future superposition of them with the tensile stresses in service of the same value. 

As far as hardnesses are concerned, it was expected that in the plastic deformed zones, the values would be higher than in the undeformed zone. However, it is found that towards the edges of the part, the hardness values are lower, which suggests that here the degree of plastic deformation is lower. It is also noted that in the flat area in the immediate vicinity of the plastically deformed area, the hardness values are close to those achieved in the curved area, which indicates that significant plastic deformation also occurs in the flat area, where the residual stresses were also determined with one of the electrotensometric transducers. 

## Figures and Tables

**Table 1:** Chemical composition of aluminum samples used for determinations/tests.

Element Concentration						
**Al [%]**	**Si [%]**	**Fe [%]**	**Cu [%]**	**Mn [%]**	**Mg [%]**	**Zn [%]**
93.85	0.122	0.629	<0.0030	0.461	4.70	<0.0010
**Cr [%]**	**Ni [%]**	**Ti [%]**	**Be [%]**	**Ca [%]**	**Li [%]**	**Pb [%]**
0.113	<0.020	0.0410	<0.0001	0.0187	0.0004	<0.001

**Table 2:** Constants $\bar{a}$ and $\bar{b}$ as a function of the h/D ratio, from standard E837.

Depth/D, h/D	$\bar{a}$ for D_0_/D = 0.35 (0.37)	$\bar{b}$ for D_0_/D = 0.35 (0.37)
0	0	0
0.05	0.037	0.069
0.1	0.081	0.159
0.15	0.115	0.239
0.2	0.137	0.299
0.25	0.147	0.339
0.3	0.151	0.364
0.35	0.151	0.379
0.4	0.149	0.387

**Table 3:** Constant values $\bar{a}$ and $\bar{b}$ as a function of the *h*/*D* ratio.

Depth [mm]	h/D	$\bar{a}$	$\bar{b}$
0	0	0.0001	0.0001
0.2	0.039	0.022	0.055
0.4	0.078	0.046	0.120
0.6	0.117	0.068	0.185
0.8	0.156	0.086	0.245
1	0.195	0.100	0.294
1.2	0.234	0.109	0.330
1.4	0.273	0.113	0.354
1.6	0.312	0.113	0.368
1.8	0.351	0.112	0.376
2	0.390	0.111	0.385

## Data Availability

Data are contained within the article.
